# Epidemiology of malaria in an area of seasonal transmission in Niger and implications for the design of a seasonal malaria chemoprevention strategy

**DOI:** 10.1186/1475-2875-12-379

**Published:** 2013-10-30

**Authors:** Julia Guillebaud, Aboubacar Mahamadou, Halima Zamanka, Mariama Katzelma, Ibrahim Arzika, Maman L Ibrahim, Elfatih Ab Eltahir, Rabiou Labbo, Pierre Druilhe, Jean-Bernard Duchemin, Thierry Fandeur

**Affiliations:** 1 Unité de Parasitologie, Centre de Recherche Médicale et Sanitaire, 634 Bd de la Nation, BP 10887, YN034 Niamey, Niger; 2 Unité de Virologie, Institut Pasteur de Madagascar, BP 1274, Ambatofotsikely, Antananarivo 101, Madagascar; 3 Parsons Laboratory, Massachusetts Institute of Technology, Cambridge, MA 02139, USA; 4 Malaria Vaccine Development Laboratory, Institut Pasteur, and Vac4all initiative, Paris, France; 5 Australian Animal Health, Laboratory of CSIRO, (AAHL), Portarlington Road EAST 28, Geelong, VIC 3220, Australia; 6 Unité de Parasitologie Médicale, Centre International de Recherches Médicale de Franceville, BP 769, Franceville, Gabon

**Keywords:** Malaria, Niger, Chemoprevention, Seasonality, Slide positivity rate, Incidence, Prevalence

## Abstract

**Background:**

Few data are available about malaria epidemiological situation in Niger. However, implementation of new strategies such as vaccination or seasonal treatment of a target population requires the knowledge of baseline epidemiological features of malaria. A population-based study was conducted to provide better characterization of malaria seasonal variations and population groups the most at risk in this particular area.

**Methods:**

From July 2007 to December 2009, presumptive cases of malaria among a study population living in a typical Sahelian village of Niger were recorded, and confirmed by microscopic examination. In parallel, asymptomatic carriers were actively detected at the end of each dry season in 2007, 2008 and 2009.

**Results:**

Among the 965 presumptive malaria cases recorded, 29% were confirmed by microscopic examination. The incidence of malaria was found to decrease significantly with age (p < 0.01). The mean annual incidence was 0.254. The results show that the risk of malaria was higher in children under ten years (p < 0.0001). The number of malaria episodes generally followed the temporal pattern of changes in precipitation levels, with a peak of transmission in August and September. One-thousand and ninety subjects were submitted to an active detection of asymptomatic carriage of whom 16% tested positive; asymptomatic carriage decreased with increasing age. A higher prevalence of gametocyte carriage among asymptomatic population was recorded in children aged two to ten years, though it did not reach significance.

**Conclusions:**

In Southern Niger, malaria transmission mostly occurs from July to October. Children aged two to ten years are the most at risk of malaria, and may also represent the main reservoir for gametocytes. Strategies such as intermittent preventive treatment in children (IPTc) could be of interest in this area, where malaria transmission is highly seasonal. Based on these preliminary data, a pilot study could be implemented in Zindarou using IPTc targeting children aged two to ten years, during the three months of malaria transmission, together with an accurate monitoring of drug resistance.

## Background

Niger (area 1,267,000 km^2^) has 15 million inhabitants, most of whom live permanently in the Sahelian zone. Malaria is the principal public health problem in this country, well ahead of meningitis, respiratory infections and diarrhoeal diseases. It is the leading cause of morbidity and mortality in children under the age of five years (more than three million subjects) and pregnant women (about 700,000 subjects) [1].

Over the last decade or so, Niger has implemented complementary malaria control strategies based on WHO recommendations, including the distribution of insecticide-treated bed nets (ITNs) and the implementation of intermittent presumptive treatment (IPT) in pregnant women [2]. Artemether-lumefantrine combinations have been used as the first-line treatment for malaria since 2005, and free healthcare for the under-fives was introduced in 2006. Niger benefited from the implementation in 2010 of the affordable medicines facility for malaria programme, which aims to improve access to artemisinin-based combination therapy (ACT) [3,4]. The national resources devoted to the fight against malaria in Niger have thus considerably improved but, despite the strengthening of complementary anti-malaria interventions in the field, their effects have been surprisingly less pronounced in Niger than elsewhere. The number of cases increased from 592,334 in 2000 to 3,138,696 in 2010, corresponding to an increase by a factor of 5.3 in ten years, whereas the population of Niger increased by a factor of only 1.3 over the same period [1,5,6]. The observed trends are puzzling as most of the indicators of performance for current interventions (rate of coverage for ITNs, availability and quality of the treatments applied, other measures designed to increase the capacities of healthcare staff and health cover) are generally satisfactory [7]. This paradoxical situation highlights the limitations of global strategies, which fail to take the heterogeneity of malaria and the regional epidemiological context into account. Indeed, there are considerable spatial and temporal variations in malaria transmission in Niger, characterized by a decreasing gradient of endemicity from South to North, linked to temperature variation and to the distribution of remnant water favouring the development of breeding sites for mosquitoes [8]. Malaria follows an endemic pattern with a major seasonal peak from August to October, which is particularly marked in the Sahelian area. Despite these differences in the epidemiological features of malaria across Niger, the entire country is subject to a common set of recommendations whereas these differences should be taken into account to develop better targeted, and more effective, preventive strategies. The causes for the steady increase in the number of malaria cases reported in Niger over the last decade are likely multifactorial and a critical evaluation of the strategic choices, and their modes of implementation, is warranted. Also, with the recent changes in the epidemiological patterns of malaria in the zones at highest risk, the model is gradually changing, from an approach based solely on screening, to strategies for combating malaria in specific populations and/or regions selected in advance to maximize the efficacy of interventions [9].

In line with this new approach, seasonal malaria chemoprevention (SMC), previously described as intermittent preventive treatment in children, has proved effective for preventing malaria in children in zones of seasonal malaria transmission [10-12]. The Sahelian zone of Niger (300 to 600 mm of rainfall per year, on average) displays a malaria endemicity of various degrees of stability, with an increase in the number of cases during the rainy season and relatively high morbidity and mortality rates, particularly in children. Targeted drug-based prophylaxis (chemoprevention) of seasonal malaria due to *Plasmodium falciparum* could, therefore, be envisaged in this part of Niger, which is also the most heavily populated part of the country [13]. However, the success of such a programme would require prior knowledge of the epidemiological profile of the endemic, the age groups most at risk and seasonal variations affecting transmission rates. If transmission rates are low and unstable, SMC limited to very young infants would probably have little effect on morbidity, and only a modest impact on overall mortality, because the group at risk includes children of up to five years of age, or even older.

The epidemiological features of malaria have been well described in Mali, Senegal and Sudan, but few data are available from Niger. With the aim of exploring the feasibility of, and conditions required for improved interventions against malaria in Niger the population of a village in an environment representative of the Sahelian zone of Niger was surveyed. The exposed population was parasitologically and clinically followed for a period of over two years, taking into account short-term meteorological variations. The results presented here are complementary to the entomological data acquired previously in the same region [14,15]. Together, these findings may help in the design of new anti-malaria interventions that are better adapted to the Sahelian context. This strategy includes the vaccination, screening and treatment of asymptomatic carriers, and seasonal prophylaxis in the most vulnerable groups.

## Methods

### Study area

This study was carried out in the village of Zindarou (13°26.09 N/2°55.23 E), in a rural area in the district of Birni N’Gaoure about 100 km southeast of Niamey, in Niger, West Africa. The village may be considered representative of the Sahelian ecological zone of Niger. The climate consists of three seasons: hot and rainy in June to October, warm and dry from November to January, and hot and dry from February to May [14-16]. According to a census performed in 2006, Zindarou has a total of 522 inhabitants, who follow a traditional rural lifestyle. The principal economic activities are agriculture and husbandry. Millet is the main crop, with onions, rice and sugar cane also produced, but on a smaller scale. Zindarou is located in the lush and fertile former river basin of the Dallol Bosso, which benefits from a longer growing season than elsewhere. This area contains many large pools with vegetation and there are substantial interactions between surface and ground water, as the water table often rises to the extent that there are large flooded areas during the peak of the rainy season, from late July through early September [16]. In addition, the area is dotted with many shallow springs, which villagers use to water their vegetable gardens. These springs are natural but often contain emergent vegetation and clean, clear water. Malaria transmission is highly seasonal and this area could, therefore, be defined as a Sahelian II transmission zone, with a relatively high entomological inoculation rate (EIR) of 285 infective bites per person per year, which is a common figure in Sahelian areas. The main vectors of malaria are *Anopheles gambiae* sl. and *Anopheles funestus*[14,15]. Access to healthcare was very limited before the implementation of the follow-up. The only healthcare structure was a private health centre receiving out-patients twice per week. During the survey, basic healthcare, including pre-natal and maternity care and vaccinations through Expanded Programme of Immunization, was dispensed free of charge. A military nurse, specifically recruited for the study, was responsible for prescriptions, drugs supply and treatment administration. Biological, clinical and parasitological data were recorded continuously.

### Population study

**Figure 1:**
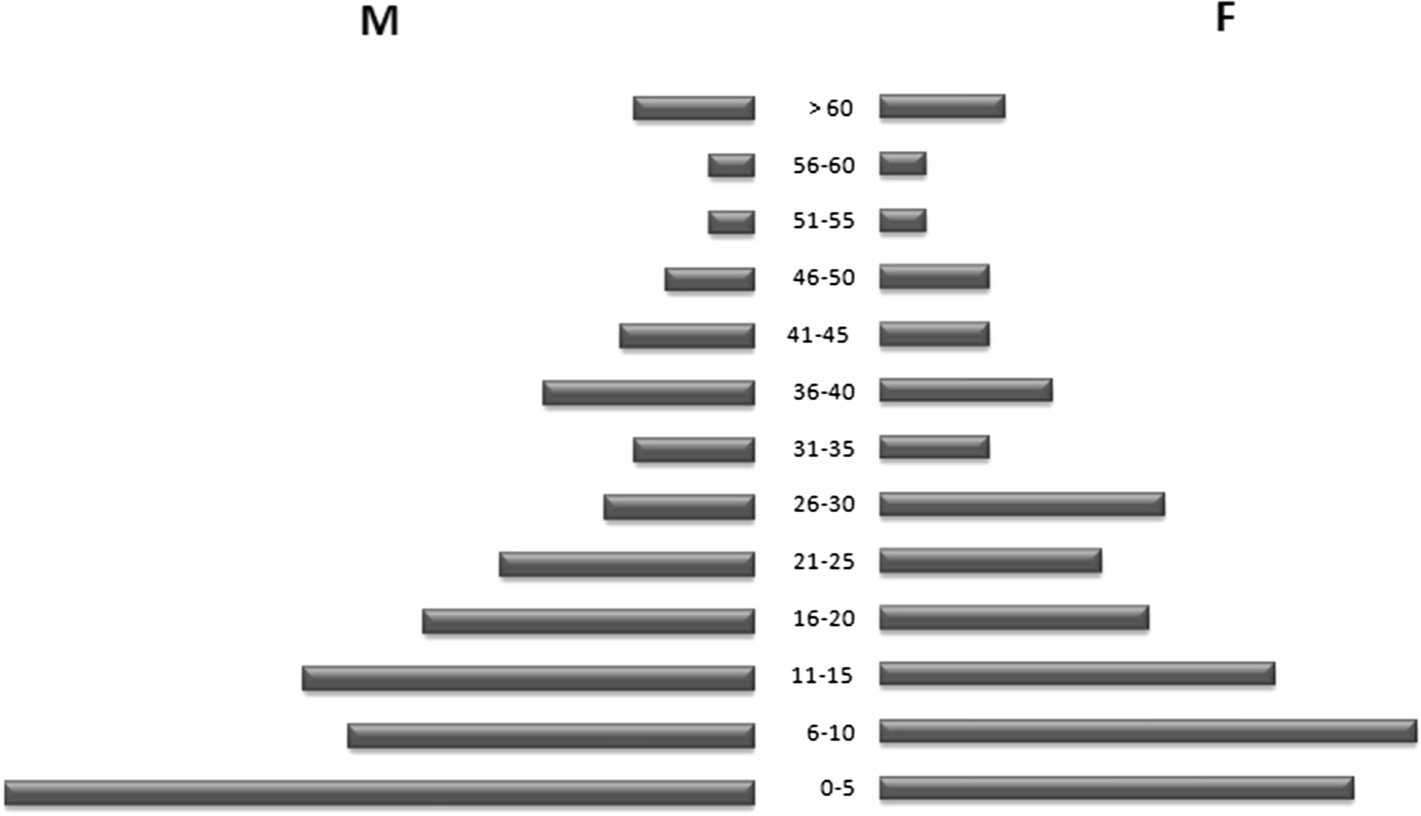
The pyramid of age in Zindarou based on the study population enrolled for the survey in 2007 (441 inhabitants).

The study was conducted from July 2007 to December 2009. All inhabitants were invited to participate to the survey, and those giving informed consent, including minors whose parents or legal guardians gave consent, were included. There were 522 inhabitants in Zindarou (Census 2006, CERMES), and a total of 441 of these individuals consented to participate in the survey. The study population was thus 441 subjects, with a mean age of 20.1 ± 17.8 years (range: 0.3 to “100 years”), 7.0% (31/441) of whom were under the age of two years, 14.6% (64/441) of whom were between two and four years of age, 14.0% (62/441) of whom were between five and nine years of age, 13.1% (58/441) of whom were between ten and 14 years of age and 51.3% (226/441) of whom were adults, defined as 15 years old or older for this population (Figure 1). The female/male ratio was 0.97 (217/224). At the beginning of the survey, each participant was assigned a unique code number, which was conserved throughout the follow-up period. Any inhabitant of the village attending the health centre was examined and, for those with code number, clinical information were recorded. An appropriate treatment was given, when necessary. Two patients requiring hospitalization were referred to the National Hospital at Niamey. All healthcare provided during the survey was free of charge for the patients.

### Passive case detection

Any villager included in the survey (N = 441) consulting at the Zindarou health centre with symptoms suggestive of malaria, including fever, was recorded as a presumptive case of malaria and was immediately treated with an ACT, in line with Ministry of Health policy. Fever was defined as an axillary temperature exceeding 37.5°C. Registration code number, name, age, sex, and temperature were noted. Thick and thin blood smears were prepared from finger-prick blood samples for each patient with presumptive malaria. The site was visited once a week by a medical supervisor for the collection of clinical information and its entry into the database. The blood smears obtained in this study were not processed locally, but were brought to laboratory facilities at Niamey on a weekly basis, for an *a posteriori* microscopic examination under standardized conditions.

### Active detection of asymptomatic carriers

Asymptomatic carriers were detected upon cross-sectional surveys performed in 2007, 2008 and 2009 on all individuals participating in the study, at the end of the dry seasons, when malaria transmission rates were considered to be the lowest. Among the 441 inhabitants of Zindarou enrolled at the start of the study, 358, 378 and 354 were available for malaria screening in June 2007, June 2008 and June 2009, respectively. For these patients, the female/male ratio was 1.01 (F = 50.1%, M = 49.9%). Axillary temperature was recorded, and thick and thin blood smears were systematically prepared for all the inhabitants of the village. The presence of the parasite was confirmed *a posteriori* at the laboratory, by microscopy examination. Individuals with a positive blood smear and an axillary temperature <37.5°C were considered to be asymptomatic carriers.

### Laboratory procedures

The blood smears were fixed and stained with Giemsa and examined by an experienced microscopist, according to standard procedure. The slide was considered positive if malaria parasites were detected in the blood smear. A blood smear was considered negative if no parasite was detected after the examination of 200 oil-immersion fields on the thick smear. When malaria parasites were detected in the blood film, parasite density was determined by counting the number of parasites present per 200 white blood cells (WBC) in the thick smear and multiplying by 40, to obtain an approximate parasite count per ml of blood. This calculation was based on the assumption of a mean WBC count of 8,000/μl of blood, considered as a representative standard value allowing calculation of the parasite count [17]. The thin smears were examined to identify the *Plasmodium* species.

### Meteorological data

A standard meteorological station was installed in Zindarou to collect data on temperature, rainfall, humidity, radiation, soil moisture, and wind. The observations were collected at high resolution (~30 minutes) and were stored using an automatic data logger. The data on temperature and rainfall are presented to shed light on the role of these meteorological variables in explaining observed seasonal variability in concurrent epidemiological data describing malaria incidence in Zindarou. The same dataset has been used to force a mechanistic model of the hydrology, entomology and malaria transmission over this village [16].

### Data processing and statistical analysis

The data collected during both longitudinal and transversal surveys were entered into two separate Excel files and processed for statistical analysis with Excel or Open Epi. Percentages, means and standard deviation were calculated. Differences in proportions were assessed in Chi-squared tests. Sample means were compared in unpaired Student’s *t*-tests. Values of *p* < 0.05 were considered significant. The positivity rate was defined as the percentage of patients with suspected malaria (“presumptive case”) proved positive by microscopy (“confirmed case”). The relative risk of malaria in the various age groups was calculated as the ratio between the proportions of subjects testing positive in two groups, using the group of under five years old as the reference group [18].

### Ethical considerations

This study was authorized by the “Health Department Direction of Dosso”, and ethical clearance was obtained from the National Ethical Review Committee of Niger. A rally was also organized, during which the purpose of the study was clearly explained to the population. Participation in the study was voluntary. All diagnosed cases of malaria were treated free of charge, in accordance with the recommendations of the Niger National Malaria Control Programme.

## Results

### Malaria morbidity

The study was carried out from July 2007 to December 2009 to assess the incidence of malaria among the 441 inhabitants of Zindarou enrolled in the study. Incidence was estimated by recording malaria cases, defined here as fever and/or clinical signs sufficiently severe for the patient to seek care at the local health unit, and being positive for asexual malaria parasites. The relationships between parasite density, fever incidence, seasonal variations, and age in the Zindarou area are not fully detailed here but presented in detail elsewhere (J Guillebaud et al., in preparation). During the passive survey, 965 presumptive malaria cases were recorded (137, 500 and 328 in 2007, 2008 and 2009, respectively). They included 280 (29.0%) that tested positive for malaria by microscopic examinations of stained blood smears (Table 1). The median age of patients consulting with malaria-like symptoms was 20.9 ± 17.4 years. There were more female than male patients (58.4% (564/965) *vs* 41.6% (401/965); *p* < 0.0001, Chi-squared test). The proportion of febrile patients testing positive by microscopy significantly varied according to age (p < 0.0001, Chi-squared test). Patients under the age of five years were more frequently diagnosed with uncomplicated malaria than patients from older age groups; the rate of malaria confirmation then decreased with age from 56.7% for children under the age of five years to 15.8% for individuals over the age of 15 years. The mean number of malaria cases per person also varied with age group, the youngest children displaying more malaria episodes than older individuals, 0.9 and 0.38, respectively. In particular, the number of malaria episodes decreased sharply after the age of ten, most likely due the development of immunity to malaria. *P. falciparum* was found to be responsible for 99.6% of infections and *Plasmodium ovale* was detected only once (0.4% of cases). No *Plasmodium vivax* or *Plasmodium malariae* cases were observed. Mean asexual parasite densities detected in confirmed malaria cases (ranging from 4, 027 to 19, 084 parasites per μl of blood) varied significantly according to the various age group (p = 0.002, Brown-Forsythe Test one-way ANOVA) but did not show any specific trend. A significant difference was however observed between mean parasite densities in individuals less than 5 years old (18, 974 parasites per μl of blood, CI95%: 13853–24095) and those above 5 years old (6, 584 parasites per μl of blood, CI95%: 3450–9720) (p = 0.0001, Student’s t-test). High parasite densities are associated with more severe infection. The mean annual malaria incidence estimated from the collected data was 0.254. The incidence of malaria was found to increase with age until the age of five years, then gradually decreased to the age of 15 years and remaining stable in adults thereafter. Table 1 shows the risk of malaria in various age groups relative to the risk of malaria in adults (≥15 years). Over the study period, the relative risk of malaria by age group compared to risk of malaria in adults was 2.88 for children under the age of two years, 3.98 for those aged two to four years and 1.74 for those aged five to nine years (p < 0.0001). The relative risk for those between ten and 14 years was not significantly different to that for adults.

**Table 1:** Repartition of malaria confirmation rate, incidence, parasites densities and relative risk by age group in Zindarou, July 2007 to December 2009

**Age group**	**Population**	**Number of**	**Number of positive**	**Asexual parasite density**	**Annual**	**RR**	**p-**
**(at t = 0)**	**size**	**presumptive cases**	**slides (%)**	**(p/μl) [CI95%]**	**incidence (°/°°)**	[CI95%]*	value**
<2 years	31	60	34 (56.7)	18, 664 [8, 074–26, 738]	439	2.88 [2.45-3.39]	<0.00012
2-4 years	64	171	97 (56.7)	19, 084 [12, 847–2,5 320]	606	3.98 [3.42-4.65]	<0.0001
5-9 years	62	106	41 (38.7)	5, 312 [1, 641–8, 984]	265	1.74 [1.46-2.09]	<0.0001
10-14 years	58	85	22 (25.9)	4, 027 [1, 136–6, 918]	152	1.0 [0.81-1.23]	0.96
≥15 years	226	543	86 (15.8)	7, 846 [2, 804–12, 888]	152	1.0	-
Total	441	965	280 (29.0)	12, 358 [9, 390–15, 326]	254	-	-

* RR: Risk of malaria by age group relative to risk of malaria in adults (≥15 years).

** p-values were calculated using the Z-score on RR. A p-value < 0.05 was considered significant.

### Seasonal variations

**Figure 2:**
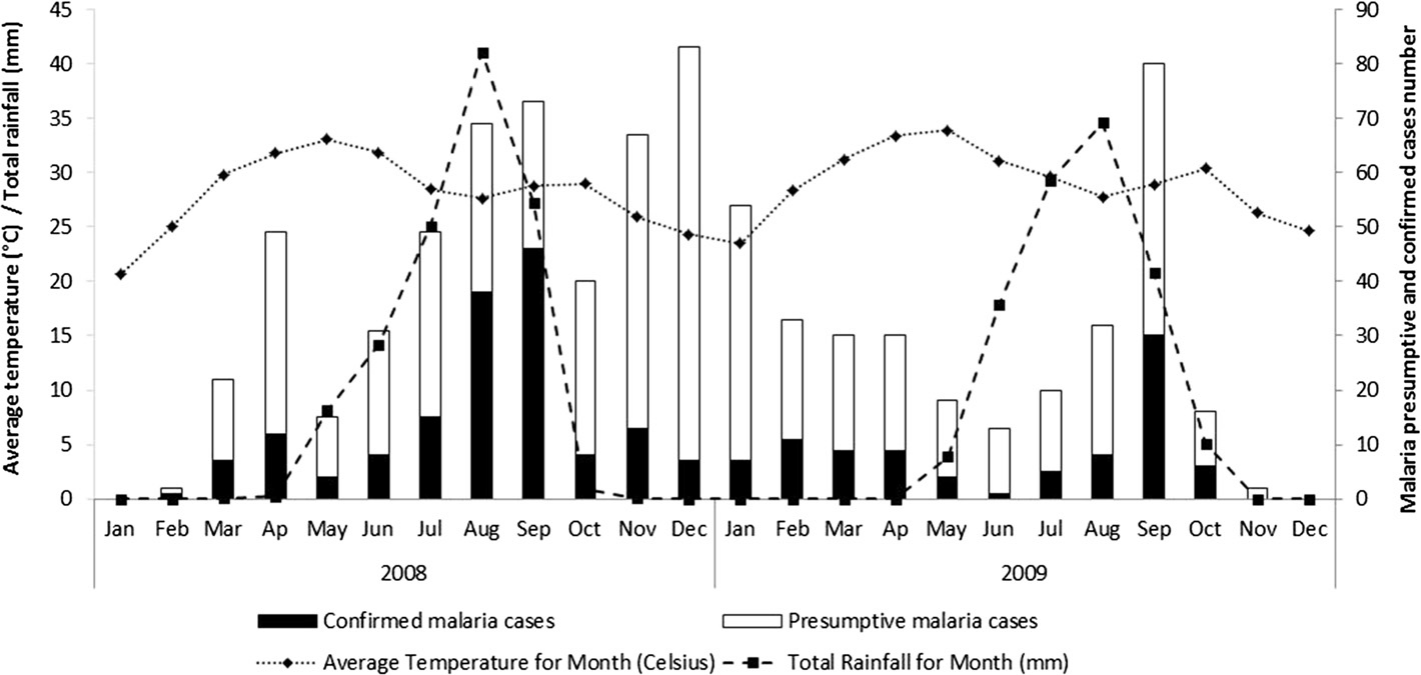
Monthly variations of malaria cases according to precipitations and temperature, Zindarou, 2008–2009.

In the Sahelian part of Niger, the dry season extends from November to May, and the rainy season from June to October. At the Zindarou site, malaria is transmitted by *An. gambiae sl* and *An. funestus,* the temporal dynamics of which are strongly influenced by seasonal variation. Mosquito abundance peaks during the rainy season (EIR of 0.78 infected bites/person/night were recorded during the rainy season in 2007) and only rare specimens, mostly of *An. funestus,* are caught throughout the dry season [15]. Figure 2 shows presumptive and confirmed malaria cases by month in 2008 and 2009. Mean temperature and rainfall were also recorded monthly. The mean temperature (Q1-Q3) was 28.5°C (20.6-33.9°C) over the study period. The coldest months were January 2008 and January 2009, with mean temperatures of 20.6 and 22.8°C, respectively; the warmest months were May 2008 and May 2009, with mean temperatures of 33.1 and 33.9°C, respectively. The mean rainfall (Q1-Q3) was 39.8 mm (0–164 mm) but the abundance of precipitation varied considerably between seasons. Rainfall levels peaked in August 2008 and 2009, at 164.3 mm and 140.1 mm, respectively, whereas there was little or no precipitation during the dry season. The dry season was slightly cooler than the rainy season in terms of the absolute mean monthly temperatures (28.0°C *vs* 29.4°C), but the difference was not significant (*p* > 0.05, t-test). By contrast, monthly cumulative rainfall was 15.5 times higher during the rainy season (899 mm) than during the dry season (58 mm), and this difference was highly significant. The incidence of malaria was higher during the first than second year of follow-up, with a monthly maximum of 46 cases of infection in September 2008; the incidence was lower in 2009, with the largest number of malaria cases in any given month being 30, in September. It is difficult to determine whether this difference reflects a study effect or short-term fluctuations of meteorological conditions. The mean number of malaria cases recorded monthly was 2.4 times higher in the rainy season than in the dry season. Most cases occurred between July and October, which can therefore be considered the target period for the implementation of preventive strategies in the Sahelian fringe of Niger. The mean parasite densities tended to increase as the incidence increased, *i.e.* mainly during the rainy season between July and October (data not shown). Indeed, changes in the numbers of malaria episodes broadly followed the temporal pattern of changes in precipitation, with a lag of 15 to 30 days between major rainfall episodes and malaria cases. This pattern presumably reflects longitudinal fluctuations of the entomological parameters associated with rainfall, and led to a peak of transmission in August in both 2008 and 2009 (data not shown). In Zindarou, fluctuations in ground water level follow the seasonality of rainfall with a lag of about one to two months [16].

### Asymptomatic and gametocyte carriage

Table 2 shows the cumulative proportion and age distribution of positive slides, for all parasitic forms and gametocytes, identified by active detection of asymptomatic carriers at the end of the three dry seasons, from 2007 to 2009. Overall, 1090 blood smears were prepared and examined and 16.0% (174/1090) were found to be positive, with only slight differences between calendar years: 18.7% (67/358), 11.4% (43/378) and 18.1% (64/354), in 2007, 2008 and 2009, respectively. All but one of the infections were due to *P. falciparum*. The exception, in 2009, was due to *P. malariae* (this case was excluded from the subsequent analyses). Asexual forms were the most frequently detected (90.2%; 157/174), and a small number of individuals were found to carry either sexual forms only (2.9%; 5/174) or both asexual forms and gametocytes (6.9%; 12/174). Age had a significant effect on the asymptomatic carriage of asexual forms of the parasite (*p* < 0.05, Chi-squared test). Asymptomatic carriage tended to decrease with age, with the parasite prevalence being 36.2, 29.4, 18.1 and 5.8% for the two to four, five to nine, ten to 14 and >15 years age groups, respectively. The exception to this general trend was that only 11.1% of children under the age of two years tested positive. There was no significant relationship between age and gametocyte carriage in the asymptomatic population. However, our data for gametocyte carriage should be treated with caution due to the small number of observations: the proportion of individuals carrying gametocytes was generally low. There was a trend for subjects aged 2 to 10 years old to have higher absolute gametocyte counts than other age groups, suggesting that individuals in this age group are the major reservoir at the end of the dry season.

**Table 2:** Repartition of asymptomatic malaria cases and gametocytes carriers by age group, (2007, 2008 and 2009)

**Age group**	**Population**	**Number of**	**Number of**
	**size**	**asymptomatic**	**gametocytes carriers (%)**
		**cases (%)**	
<2 years	27	3 (11.1)	1 (33.3)
2-4 years	152	55 (36.2)	7 (12.7)
5-9 years	187	55 (29.4)	5 (9.1)
10-14 years	155	28 (18.1)	3 (10.7)
≥15 years	569	33 (5.8)	1 (3.0)
Total	1090	174 (16.0)	17 (9.8)
p-value*	-	<0.05	>0.05

*p-values were calculated using Chi squared test. A p-value <0.05 was considered significant.

## Discussion

SMC requires the acquisition of up-to-date data relative to the regional epidemiological features of malaria prior to implementation, such that target populations can be identified and protocols for treatment delivery optimised. Here, we report longitudinal follow-up of clinical cases of malaria and active screening for asymptomatic carriers in the village of Zindarou for this purpose.

Malaria diagnosis and patient follow-up in Niger is based almost exclusively on a clinical examination focusing on non-specific symptoms. This presumptive diagnosis is imprecise and over-estimates the number of cases, by confusing malaria and other fevers, such as those caused by arboviruses. In Niger, the proportion of positive slides is strongly associated with the bioclimatic context. The frequency of positive slides is negatively correlated with latitude during the rainy season, whereas no relationship to longitude is found, irrespective of the transmission rate [8]. At Zindarou, the rate of confirmation for presumptive cases of malaria is only 29.0%: the rate of confirmation varied from 56.7% in children under the age of five years to 15.8% in adults (>15 years). Observations at national level and at Zindarou suggest that in the northern regions where malaria is epidemic, biological confirmation should be sought for all cases by rapid diagnostic tests; in the Sahelian zone of Niger preventive seasonal treatment should be introduced for children, to decrease the incidence of malaria and malaria-associated mortality.

Incidence decreased with age at Zindarou. This has also been observed in other Sahelian settings, indicating that this village is indeed representative of the Sahelian zone of Niger [19,20]. The under-fives were the group most at risk in Zindarou and, thus, should be the target population for SMC. The number of cases was 2.4 times higher during the rainy season than during the dry season, and most of the confirmed cases were between June and October. These seasonal variations can be used to optimise conditions for implementation of the SMC in the Sahelian part of Niger, including identification of where and when to start and finish.

Changes in the number of malaria cases broadly follow short-term variations of meteorological conditions. The lag time in Zindarou between an excess risk of malaria, linked to precipitation, and a significant increase in the number of cases, is about 30 days. A previous study in Magaria (Eastern Niger, latitude 12°59'52'' N) estimated the lag time between rain and a malaria surge to be 22 to 39 days [21]. However, only suspected cases were considered in this previous work, whereas the Zindarou study shows that changes in the incidence of malaria episodes parallels meteorological data only if confirmed cases are considered (confirmation excluding fevers of viral or bacterial aetiology). This lag is roughly the time required for the parasite to complete its life cycle in the vector and in the vertebrate host. These observations are reminiscent of reports in East Africa [22].

Active screening for asymptomatic carriers at the end of the dry season revealed that parasite carriage was more frequent in children (36.2% for children aged two to four years and 29.4% for children aged five to nine years) than in adults (individuals over the age of 15 years; 5.8%), again confirming the epidemiological relevance of the village chosen. The youngest individuals were asymptomatic carriers not only of asexual stages, but also of gametocytes. These sexual forms are responsible for transmission restarting after the end of the dry season. Asymptomatic carriers of gametocytes were found in all age groups, but were clearly over represented in the two- to ten-year age group, identifying these children as the principal reservoir for the transmission restarting with the first rains of May. A similar pattern has been found in Burkina Faso [23].

SMC involves the administration of anti-malarial drugs at pre-determined times, regardless of whether or not the treated subjects are infected. It therefore seems to conflict with WHO guidelines recommending that suspected cases of malaria should be confirmed biologically before treatment. However, by considering specific regional features when developing preventive strategies, the two approaches (biological confirmation before treatment and SMC) could prove not only to be complementary, but also synergic, increasing the efficacy of anti-malarial programmes. Since 2012, the WHO has been recommending, through its global anti-malarial programme, SMC for the seasonal transmission in the Sahelian zone [10]. SMC targets children aged three to 59 months and involves complete treatment with an AQ + SP combination administered at regular one-month intervals, beginning at the start of the season in which transmission is highest. In the epidemiological context of Zindarou, it is recommended to target children aged from two to five years and to administer treatment during the rainy season. By contrast, the more widespread use of SMC could not reasonably be envisaged to provide the same advantages in the Sudanese zone of Niger, in which malaria transmission is hyperendemic and occurs throughout the year.

Over the last 30 years, the malaria control strategies implemented have come up against the problem of the emergence and rapid propagation of parasites resistant to anti-malarial drugs, and especially artemisinin [24]. The medium- to long-term consequences of resistance to the drugs employed for SMC therefore need to be considered, especially if SMC involves the use of ACT. A recent *ex vivo* study with fresh isolates collected in south Niger showed that *P. falciparum* had retained its susceptibility to conventional antimalarials, including AQ, and to new molecules, including artemisinin derivatives [25]. Similarly, a therapeutic efficacy test in 2011 (simplified 28-day standard protocol) at the sentinel site at Gaya, with 159 children under the age of 15 years showed that artemether/lumefantrine (AL) and artesunate/amodiaquine (AA) combinations were fully effective for the management of uncomplicated *falciparum* malaria, with adequate clinical and parasitological response rates in 98.5 and 95.5% of cases, respectively (unpublished data). By contrast, the current extent of resistance to SP in Niger is only poorly documented. Before complete, intermittent treatment with AQ + SP can be considered, additional observations are required, including more recent data, providing a better description of the susceptibility of the parasite to these anti-malarial drugs. The establishment of a reliable system for monitoring drug resistance at national scale and the continuing operation of a point-of-care network to document cases and possible adverse effects of treatment are pre-requisites for SMC implementation.

Many studies have shown that SMC in young children is an effective way of preventing malaria in regions of seasonal malaria transmission [10-12]. The highly seasonal nature of the rainfall, and the observed endemicity of malaria, indicate that Niger would be a suitable country for the implementation of SMC, as in other Sahelian countries which already benefit from this approach [26-28]. In Mali, the annual incidence of malaria was halved in children between the ages of six months and ten years, by administering sulphadoxine-pyrimethamine (SP) twice, at eight-week intervals [29]. These observations and the results of a study in Senegal in a very similar epidemiological context [30], suggest that seasonal prophylaxis with SP plus artesunate (AS) or SP plus amodiaquine (AQ) (three doses, at one-month intervals, between June and September) administered to children under the age of five years in the Sahelian zone of Niger would be beneficial.

On the basis of the results presented here, a pilot study of the administration of a complete AQ + SP treatment to children between the ages of two and five years, during the months of July, August and September, could be envisaged at Zindarou. The clinical and parasitological follow-up of a selected cohort, with, in particular, close monitoring of responses to anti-malarial drugs and the observation of possible rebound effects in the following years, would make it possible to explore the technical feasibility of this approach. If feasible, and if the consequences in terms of morbidity and mortality are satisfactory, the approach could then be extended to the entire Sahelian zone in Niger.

## Competing interests

The authors declare no conflict of interest. None of the authors have financial or personal conflicts of interest related to this study. The corresponding author has full access to all data in the study and final responsibility for the decision to submit this publication.

## Authors’ contributions

JG and TF designed the study and coordinated its execution, analysed the data and wrote the first draft of the paper. PD and JBD provided guidance and field expertise, and reviewed the design of the study and the report drafts; they selected the Zindarou study site and conceived the initial protocol for the follow-up. AM supervised field work and was responsible for the supervision of data collection. HZ, MK, IA, MIL, and RL contributed to the data collection, and to field and laboratory works. AABE contributed to meteorological data collection. All authors have read and approved the final version submitted.
